# Avances en citometría de masas y aplicabilidad en patología digital para estudios clínico-traslacionales en oncología

**DOI:** 10.1515/almed-2021-0051

**Published:** 2021-08-25

**Authors:** Karina Cereceda, Roddy Jorquera, Franz Villarroel-Espíndola

**Affiliations:** Laboratorio de Medicina Traslacional, Instituto Oncológico Fundación Arturo López Pérez, Santiago, Chile

**Keywords:** imágenes obtenidas por citometría de masas (IMC), imágenes por haz de iones multiplexados (MIBI), oncología

## Abstract

El desarrollo de la citometría de masas y posteriormente su adaptación para el análisis de secciones histológicas ha revolucionado la forma de caracterizar a nivel espacial múltiples componentes de manera simultánea, permitiendo la correlación genotípica y fenotípica de la célula y su entorno durante estudios clínicos-traslaciones. En este trabajo, hemos revisado los hitos más relevantes en el desarrollo, implementación y aplicabilidad del análisis de imágenes de componentes múltiples para el estudio de cáncer y otras dolencias, y enfocado nuestro interés que aquellos autores que utilizan imágenes obtenidas mediante citometría de masas o bien haz de iones. Esta revisión tiene como objetivo que el lector se familiarice con las estrategias técnicas de verificación de la herramienta y las múltiples posibilidades de uso abordadas por diferentes autores, y además, poder proyectar sus propias investigaciones hacia la utilización de imágenes obtenidas por citometría de masas (IMC), o imágenes por haz de iones multiplexados (MIBI) en cualquiera de los campos de investigación biomédica.

## Introducción

Muchos esfuerzos se han realizado para integrar las distintas fuentes de información conocida sobre la biología tumoral, desde aspectos genéticos hasta morfológicos [1], [2], [3], [4]; sin embargo, éstos aún no han podido integrar los múltiples biomarcadores que han resultado ser prometedores en la estratificación de pacientes o bien como herramienta para la asignación de una terapia en particular, y su posterior correlación con el desenlace clínico esperado.

El microambiente tumoral se entiende como el entorno extracelular y los componente celulares propios del órgano afectado que rodean a las células tumorales. Este ambiente consiste de células neoplásicas y no neoplásicas como, fibroblastos, linfocitos, macrófagos y otras células inmunes, así mismo componentes vasculares, y la matriz extracelular propiamente tal, considerando sus fluidos y moléculas disueltas tales como citoquinas secretadas, quimioquinas, metabolitos y vesículas extracelulares [5], [6], [7]. Actualmente, existe abundante literatura para sugerir que las células no neoplásicas y que residen en el vecindario tumoral pueden jugar un rol activo en el proceso neoplásico e incluso facilitar la evasión del sistema inmune por parte de la célula tumoral [7]. En cuyo escenario, el microambiente tumoral puede promover procesos de transformación y progresión maligna, metástasis e incluso resistencia a terapias convencionales y emergentes [6, 8].

Con el paso de los años, los problemas de salud en países desarrollados han cambiado, la población ha envejecido y por tanto son cada vez más frecuentes las condiciones crónicas multi-patológicas, así mismo el cáncer.

La investigación basada en la evidencia y los adelantos tecnológicos, han permitido que los centros de salud modernos evolucionen hacia modelos más holísticos en el cuidado de la salud, priorizando la atención centrada en el paciente [9] y promoviendo una estratificación de pacientes basada en mejores estrategias diagnósticas o mejores herramientas predictivas de éxito terapéutico, en particular en pacientes oncológicos.

Según la Sociedad Americana de Oncología Clínica (ASCO), pan-ómica (“pan-omics”, en inglés), es conocida como la integración de datos clínicos con aquellos provenientes de diferentes plataformas analíticas u ómicas [10]. Esta estrategia de integración ha sido identificada como uno de los impulsores clave que moldearán el futuro de la atención en cáncer para el 2030 [11],

La implementación de ciencias “ómicas” en oncología [12, 13], ciencia de datos en registros clínicos electrónicos [14] y más recientemente, la integración de “radiómica”, patología digital, bioinformática e inteligencia artificial [15], entre otros avances tecnológicos en medicina, han permitido que hoy sea posible, la estratificación de poblaciones de pacientes en subpoblaciones que difieren, en la susceptibilidad a una patología o en la respuesta a un tratamiento en particular.

## Imágenes de múltiples componentes y espectrometría de masas

Recientemente, avances en el análisis espacial unicelular y multiplexado de tejidos nos permite observar la complejidad de la biología del cáncer con una resolución sin precedentes [16]. Hoy es posible identificar diferentes tipos y estados celulares en un tumor y su ubicación espacial exacta dentro de éste. Todo esto, junto con la biología computacional, permitiría entender de mejor manera la evolución y heterogeneidad tumoral dentro de su microambiente, construir modelos que abarquen la complejidad de los datos pan-ómicos en distintos pacientes con la promesa de perfeccionar el diagnóstico y la terapéutica [11, 17, 18].

La heterogeneidad en las respuestas de los pacientes con cáncer a las inmunoterapias ha hecho evidente que es necesaria una mayor comprensión del microambiente tumoral [19] y las interacciones celulares que los constituyen. Esto representa una oportunidad para el desarrollo de nuevas tecnologías y métodos que permiten obtener datos ómicos e imágenes multiplexadas de muestras de tejido [20] y descubrir potenciales blancos terapéuticos [17, 21, 22].

A todas luces, la continua utilización de la inmunohistoquímica (IHQ) a nivel clínico, junto con diversas herramientas de captura de imágenes disponibles, enfatizan la importancia de la información espacial para una patología digital capaz de rendir a las demandas y necesidades de la investigación clínico-traslacional.

En la práctica clínica, el protocolo estándar recomendado para el diagnóstico de muchos tipos de cáncer incluye un examen microscópico de una muestra histopatológica fijada y teñida utilizando métodos inmunohistoquímicos en un portaobjetos [23, 24].

La IHQ convencional, además de mostrar resultados con una alta variabilidad inter-observador [25], posee una limitación técnica importante, ya que sólo permite teñir una sección de tejido con dos o tres marcadores a la vez. Esto implica que el estudio de múltiples biomarcadores generalmente requiera el análisis de múltiples secciones mediante cortes histológicos seriados, algo complejo en biopsias con pequeñas cantidades de material disponible [24]. Además, esta estrategia limita la posibilidad de generar una visión global de un microambiente inmunológico complejo. Por ejemplo, subtipos de células T CD8 positivas que infiltran el nido tumoral y que presentan niveles funcionales diferenciales (apoptóticas, proliferativas, citotóxicas, de memoria, etc.), o bien identificar la diversidad de poblaciones linfocitarias en una misma región de tejido tales como CD8, CD4, y CD20 [24, 26]. Por otra parte, la expresión de ciertas moléculas, como PD-L1 en la superficie de células tumorales es comúnmente utilizado para la asignación de terapias modernas en múltiples neoplasias [27, 28], y la presencia de infiltrado inflamatorio de tipo linfocítico así como la abundancia de PD-1 en la superficie de las células T CD8 positivas, han sido fuertemente demostradas por predecir la capacidad de respuesta a tratamientos inmuno-oncológicos que bloquean la unión PD-L1 / PD-1 [22]. Todos estos marcadores pueden ser predictivos individualmente o en combinación, y algunos casos ya se plantea la necesidad de considerarlos en su conjunto por el valor pronóstico en varios tipos de cáncer [20, 22].

Diferentes técnicas basadas en IHQ han sido desarrolladas para poder teñir y visualizar un mayor número de moléculas en la misma muestra, éstas técnicas se conocen comúnmente como IHQ multiplexada y si bien en combinación con anticuerpos secundarios unidos a reporteros fluorescentes, han sido un gran aporte al conocimiento, éstas técnicas tienen importantes inconvenientes como la interferencia espectral al usar varios marcadores, la reactividad cruzada entre los anticuerpos, el fotoblanqueo o pérdida de señal fluorescente, y la auto-fluorescencia inherente del tejido [29].

Varios métodos recientes logran una mayor complejidad analítica en la detección de múltiples componentes, pero a menudo con menor sensibilidad, rendimiento o accesibilidad [30]. En la Tabla 1, se presenta un resumen de diferentes tecnologías y procedimientos aplicados mayormente a tejidos fijados y que permiten la obtención de imágenes de componentes múltiples.

**Tabla 1:** Resumen de características de plataformas con tecnologías de detección multiplexada (>10 blancos).

Método / tecnología	Autoría	Tipo de tejido	Analito	Técnica de detección	Multiplexado	Resolutión	Ventajas	Debilidades	Descriptión
Microscopía de fluorescencia multiplexada (MxlF)	No privada [31]	Tejido FFEP [31]	Proteínas y ARN [32]	Fluorescencia [32]	61 proteínas [31, 32]	Subcelular, ∼1 μm [32]	Generación de imagenes multiplexadas [32]	Realineamiento de imágenes obtenidas por cada ronda de adquisición de imágenes [32]	Método de microscopía de fluorescencia multiplexada (MxlF) para la caracterización cuantitativa, unicelulary subcelular de multiples analitos en tejido FFEP. La inactivación química de los tintes fluorescentes despuís de cada ronda de adquisición de imágenes en ciclos iterativos de tinción e imagen [31]
Expresión génica unicelar (10X Chromium)	10X Genomics	Tejido FFEP o congeladas [19]	ARN [19]	ADN con código de barrasy NGS [19]	Decenas de miles de transcritos	Celular [19]	Transcriptoma completo [19]	Sin resolución bidimensional [19], no hay generación de imágenes multiplexadas	Método que combina el análisis de expresión génica unicelular, con la detección decientosde protefnasde la superficie celulara alta resolución para una citometria multiomíca (hoja de producto)
Expresión génica espacial (10X Visium)	10X Genomics	Tejido FFEP o congeladas [19]	ARN [19]	ARN con código de barrasy fluorescencia [19]	Decenas de miles de transcritos	Celular, 55–100 μm [19]	Transcriptoma completo [19] Generación de imágenes multiplexadas	Diferentes tipos celulares pueden ser captados en una región con código de barra [19]. Baja resolución	La tecnología Visium combina el análisis de expresión genica con tinción e imágenes por inmunofluorescencia para obtener una caracterización multiómica dentro de un contexto espacial (hoja de producto)
InsituPlex	Ultivue	Tejido FFEP o congeladas [19]	Prot eínas y ARN [20]	ADN con código de barrasy fluorescencia [19]	16 proteínas [20]	Subcelular [19]	Generación de imágenes multiplexadas [19]	No compatible con scanners automatizados de láminas [19]	La tecnología InsituPlex utiliza amplification y códigosde barras deADN conjugados con anticuerpos primarios para proporcionar imágenes bidimensionales multiplexadas en portaobjetos completos [19]
Codetección por indexación (CODEX)	Akoya	Tejido FFEP o congeladas [19]	Protetínas [19]	ADN con código de barrasy fluorescencia [19]	50 proteínas [16, 33]	Subcelular, ∼260 nm [20]	Generación de imágenes multiplexadas [19]	Puede consumir mucho tiempo [19]	Anticuerpos conjugados con secuentias de oligonucleotidós únicas se detectan de forma cíclica mediante extensión secuencial del cebador con nucleótidos marcados con fluorescencia [16]
Perfilamiento espacial digital (DSP)	Nan oSt ring	Tejido FFEP o congeladas [19]	Prot eínas y ARN [19, 32]	ADN con código de barras y Luz ultra violeta [20, 32]	40 proetínas o más de 90 ARN [16, 32]	Celular, ∼10 μm [19, 32]	Transcriptoma completo y alto nivel de automatización [19]	Sin generación de imágenes multiplexadas [19] Consume mucho tiempo y baja resolución [16, 28]	Método que etiqueta anticuerpos osondas deARN con oligonucleótidos de ADN fotoescindibles que se liberany contabilizan después de la exposión ultravioleta en regiones de interés espetificos del tejido [10, 16]
Imágenes multiplexadas con haz de iones (MIBI)	lonPath	Tejido FFEP [34]	Proteínas y ARN [32]	Metales Lantánidos y espectrometría de masa [32]	36 proteínas [32, 35]	Subcelular, ∼200 nm [32]	Generación de imágenes multiplexadas con alta resolucion [16]	Costosoy consume mucho tiempo [16]	Método que combina anticuerpos conjugados con isótopos de lantánidos y detección a través de un espectrómetro de masas equipado con una fuente de iones de oxígeno [16, 35]
Imágenes con citometria de masas (IMC)	Fluidigm	Tejido FFEP o congeladas [19]	Proteínas y ARN [32]	Metales Lántanidos y espectrometría de masa [32]	32 proteínas [32]	Subcelular [19], ∼μm [32]	Generacion de imagenes multiplexadas [36].	Costosoy consume mucho tiempo [16, 36]	Tecnología que utiliza un láser pulsado para realizar la ablación de una sección detejido. Anticuerpos conjugados a isótopos de lantánidos se detectan a través de un espectrómetro de masas [16, 37]
Inmunotinción con amplificación de señal por reactión de intercambio (Immuno- SABER)	No privada (BO)	Tejido FFEP o congeladas. Preparaciones celulares [30]	Proteínas [30]	ADN con código de barrasy fluorescencia [30]	10 proteínas [30]	Subcelular, ∼160 nm combinando Microscopia de expansión [30]	Compatible con varias muestrasy plataformas. Generación de imágenes multiplexadas con alta resolución [30]	Dilución de las señales de fluorescencia al combinar con microscopía de expansion [30]	Múltiples anticuerpos primarios con códigos de barras de ADN, se hibridan con ADN monocatenariosgenerados mediante reacciones de intercambio de cebadores [16].
Slide-seq	No privada [38]	Tejido FFEP o congeladas [38]	ARN [38]	ADN con código de barrasy NGS [38]	Decenas de miles de transcritos [38]	Celular, ∼10 μm [38]	Transcriptoma completo asociado a coordenadas en tejido [16]	Baja resolucion	Método que transfiere ARN de las secciones del tejido a una superficie cubierta de perlas con códigos de barras de ADN en posiciones conocidas del portaobjetos, lo que permite inferior laubicación del ARN secuenciado [38].

ADN, ácido desoxirribonucleico; ARN, ácido ribonucleico; FFEP, tejidos fijados en formalina y embebidos en parafina.

Algunos de estos nuevos métodos están basados en inmunofluorescencia cíclica [31], el uso de oligonucleótidos como códigos de barra [19, 30, 32, 33, 38, 39], espectrometría de masas combinada con técnicas histológicas clásicas y alternativamente, espectrometría de masas dirigida basada en anticuerpos conjugados [34, 37]. Estas nuevas aplicaciones tecnológicas permiten obtener resultados con una mayor resolución fenotípica en comparación a métodos inmunohistoquímicos clásicos. Además, permiten la optimización de la muestra disponible, mediante la detección simultánea de múltiples marcadores utilizando una misma sección de tejido; e incluso, aumentar la sensibilidad de un método al combinar más de un marcador para un mismo tipo celular específico.

**Figura 1:**
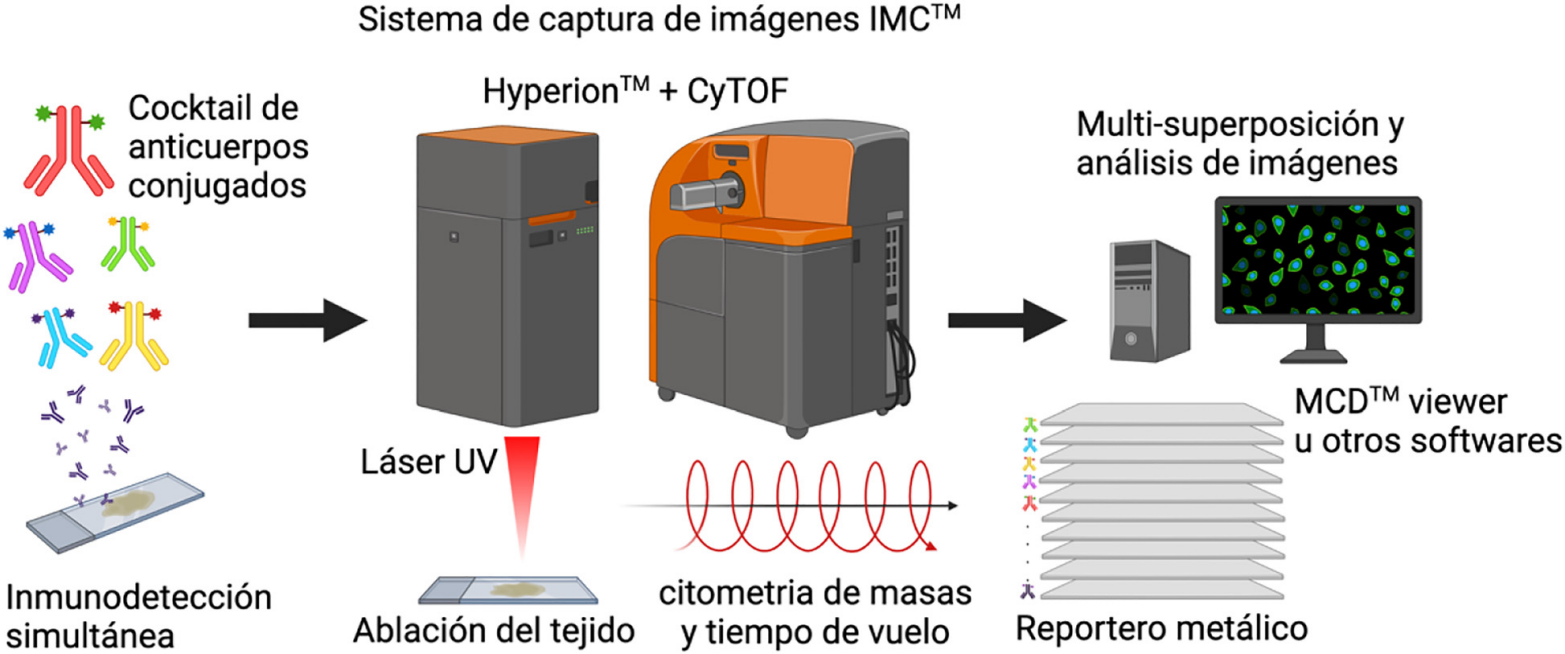
Flujo de trabajo de imágenes obtenidas por citometría de masa (IMC™). Cortes histológicos de tejidos o células inmovilizadas son inmunodetectados simultáneamente con múltipes anticuerpos conjugados con isotopos diferentes (reporteros). La ablación del tejido mediante pulso de láser ocurre en Hyperion™ y la muestra vaporizada es conducida al detector de masas del citómetro de masas (CyTOF®) mediante un flujo de plasma de gas inerte. Un set de coordenadas permite la reconstrucción del tejido y su asociación con las abundancias de cada reportero. Diferentes herramientas permiten análisis y superposición de señales (imágenes). Figura diseñada por https://app.biorender.com/.

Respecto de las imágenes de citometría de masas, éstas son obtenidas a través de un análisis de tiempo de vuelo de elementos metálicos poco abundantes que han sido conjugados a un anticuerpo específico, de este modo utilizando el principio descrito para la citometría de masas, cada anticuerpo utilizado actúa como detector y reportero simultáneamente [40] (Figura 1).

La detección de múltiples blancos es posible dada la existencia de isótopos estables derivados de la familia de los lantánidos, los cuales son quelados mediante un polímero sintético que contiene los grupos funcionales 1,4,7,10-tetraazaciclododecano-1,4,7,10-tetraacetico (DOTA) o dietileno triamino pentaacetico (DTPA), los cuales se conjugan a la cadena pesada de la Inmunoglobulina G (IgG) a través de grupos sulfhidrilos activados. Cerca de 40 isotopos han sido ampliamente utilizados corriente, los cuales se resuelven con una precisión de una unidad de masa atómica en más del 95% de los casos y para los cuales existe un protocolo de conjugación optimizado [41].

Del punto de vista de la adquisición de las imágenes, la tecnología imágenes obtenidas por citometría de masas (IMC) permite la ablación del tejido o células inmovilizadas sobre un cristal de silicato convencional mediante un pulso de láser de 213 nm y con un foco de 1 µm de diámetro. La muestra es vaporizada en cada pulso y conducida al detector de masas del citómetro mediante un flujo de plasma de un gas inerte. Cada pulso de láser genera además de la nube de átomos, un set de coordenadas que permiten la reconstrucción del tejido y su asociación con las abundancias de los reporteros en cada evento [40].

Por otro lado, imágenes por haz de iones multiplexados (MIBI) es otra alternativa que permite la caracterización de secciones histológicas basado en anticuerpos marcados con metales [34]. El principio es muy similar al IMC, con la diferencia en las condiciones de ablación, en ese sentido, el MIBI utiliza un haz de iones de oxígeno en una cámara al vacío, al contrario del sistema IMC que utiliza un láser en una cámara a presión atmosférica. En términos muy generales, ambas tecnologías parecen ser homologables y comparables en resultados de sensibilidad, resolución, complejidad de resultados [42]. Estos métodos, en algunos casos complementados con otras tecnologías como transcriptómica de resolución celular, podrían permitir obtener valiosa información de la complejidad biológica presente en el microambiente tumoral.

En la siguiente sección revisaremos los principales hitos en la evolución tecnológica de la captura de imágenes de múltiples componentes tanto IMC y MIBI, y su aplicación en investigación clínico-traslacional.

## Cronología en el análisis de imágenes de componentes múltiples

El análisis de imágenes obtenida mediante espectrometría de masas ha aportado con información suficiente para proponer modelos terapéuticos e incluso diagnósticos que podrían permitir la segregación de pacientes de acuerdo a patrones genotípicos y fenotípicos de la célula tumoral, o bien mediante las características arquitectónica del entorno peritumoral. En la actualidad, diferentes grupos de científicos, académicos y clínicos trabajan con herramientas para segmentación de tejido y tipificación del fenotipo celular en muestras histológicas, tanto al diagnóstico, como al inicio de un tratamiento, e incluso a distintos momentos de la ruta terapéutica del paciente (Tabla 2).

**Tabla 2:** Resumen de estudios basados en imágenes multiplexadas mediante reporteros metálicos y citometría de masas.

Tipo de estudio	Primer autor	Patología/tejido de interés	Número de marcadores	Objetivo
Optimizatión de la técnica	Angelo, Michael [34]	Cáncer de mama	10	Validación de MIBI en muestras de FFEP con aplicación clínica.
	Gerdtsson, Erik [43]; Bath, Izhar [61]; Giesen, Charlotte [37]	Diferentes tipos de tumores	18 a 32	Validación de la técnica IMC para la diferenciación de tipos celulares especificos.
	Martinez-Morilla, Sandra [57]	Melanoma	26	Validación de la plataforma “AQUA” para analisis de IMC Identifiación de 10 candidatos a biomarcadores que responden a inmunoterapia. Confirmación de B2M como bianco asociado a supervivencia.
	Schulz, Daniel [45]	Cáncer de mama	3 ARNm, 16 proteínas	Valdicación de detección simultanea de nuceótidos y proteínas mediante IMC en ensayos de célula individual. Determinatión de una alta correlación entre los niveles de ARNm y proteína para HER2 pero no para CK19. CXCL10 se expresa en agrupación en celulas del estroma y cuya expresión se correlaciona con la presencia de células T.
	Guo, Nannan [62]	Tejido intestinal de fetos y adultos	34	Desarrollo y validación de panel de 34 anticuerpos para análisis por IMC de muestras congeladas.
Caracterización del microambiente tumoral	Carvajal-Hausdorf, Daniel [52]; Rost, Sandra [63]	Cáncer de mama	3 a 18	Caracterización de los niveles yfunciones de HER-2 en Cáncer de mama.
	Keren, Leeat [35]; Ptacek, Jason [60]	Diferentes tipos de tumores	15 a 36	Expresión y localización de diversas proteínas inmuno reguladoras en diferentes tipos de tumores
	Ijsselsteijn, Marieke E [47]	Cáncer colorectal	40	Descripción de panel de anticuerpos para monitoreo del microambiente inmune del tumor mediante IMC.
	Li, Ran [64]	Carcinoma de células escamosas (pulmón)	21	Infiltración en el tumor de células T CD45RO+CD8+. Descripción de una nueva población de células T CD3-CD4+ en el microambiente inmuno tumoral (TIME).
	Singh, Nikhil [49]	Riñón	23	Desarrollo de un Atlas de marcadores de tejido de riñón. Identificación de posibles nuevos tipos celulares que permiten diferenciación de tejido normal y anormal.
Misceláneo patología digital	Theil, Diethilde [48]	Nódulos linfoides de monos cynomolgus	11	Caracterización de subpoblaciones linfocitarias. Tratamiento con ofatumumab genera una población rara de células T que son CD3+, CD8+, CD20+, localizada en la periferia de folfculos de células B.
	Wang, Chong [65]	Pulmón, intestino, bazo, hígado y riñón de pacientes fallecidos de COVID-19	23	Infiltración de macrófagos CDllb+y células dendriticas CDllc+en pulmones e intestinos. Altos niveles de de IL-10 en pulmones e intestinos; Sobreproducción deTNF-α en pulmón, intestino, riñón y bazo.

B2M, beta2-microglobulin; CK19, citoqueratina 19; FFEP, fijados en formalina y embebidos en parafina.

## Citometría de masas para histología

En 2014, Giesen y colaboradores reportan la utilidad de la citometría de masa acoplado a tiempo de vuelo (CyTOF) utilizada para el análisis de muestras en suspensión, como herramienta para la recolección de imágenes multiparamétricas utilizando la misma tecnología de CyTOF. Este primer trabajo, se realizó en lesiones malignas de mama y sus respectivos controles no-tumorales, donde cada anticuerpo fue marcado covalentemente al isotopo metálico de detección, permitiendo analizar 32 proteínas individualmente a resolución subcelular. La construcción de una imagen de alta dimensión (*high-dimensional image*) comprende la superposición de los tiempos de vuelo de cada reportero y los registros de coordenadas del láser de ablación. De esta manera se logra una composición multidimensional y multiparamétrica con resolución espacial suficiente para llevar a cabo estudios de segmentación y fenotipo a nivel de tejido [36]. Previamente los autores evaluaron mediante inmunodetección clásica, la concordancia entre los patrones e intensidades de tinción para cada anticuerpo, así también establecieron las variaciones obtenidas entre anticuerpos conjugados con el metal reportero y aquellos no conjugados.

En general, se observó un rango entre 2–27% de variabilidad en la señal, pero no en el patrón de la marca, esta diferencia fue atribuida a las variaciones propias de tinciones histológicas en secciones seriadas no idénticas [37]. Mediante análisis computacional y bioinformático, las imágenes obtenidas por citometría de masas (IMC) probaron ser altamente sensibles, reproducibles y análogas a las obtenidas por herramientas clásicas de inmunohistoquímica, inmunocitoquímica e inmunofluorescencia, e incluso permiten distinguir estructuras y características celulares individuales y del tejido completo.

El uso de IMC para el análisis de células individuales tuvo su revolución con el trabajo de Gerdtsson y colaboradores [43], quienes integraron al análisis de célula única de alta definición (HD-SCA) [44] junto con las imágenes procedentes de la citometría de masas, permitiendo el análisis morfológicos y fenotípico de células raras únicas, de células tumorales circulantes e incluso células tumorales diseminadas y enfermedad mínima residual. Mediante líneas celulares derivadas de cánceres humanos (LNCap en próstata y MDA-MB-231 en mama), los autores establecieron las condiciones de sensibilidad, especificidad y linealidad de las detecciones de estas fracciones celulares dentro de una matriz de sangre humana entera. La identificación de la célula tumoral dentro del extendido leucocitario, se realiza mediante tinción fluorescente con CD45 y Citoqueratina, y posteriormente se realiza el marcaje con los metales reporteros y la batería de anticuerpos establecida. Basado en las coordenadas de localización espacial tomadas previamente, los autores realizaron capturas de imágenes en una región de interés de 400 × 400 um y una resolución de 1um2 por pulso de laser de 200 Hz. Sin la necesidad de un panel de anticuerpos muy extenso, la calidad de imagen obtenida por IMC permitió a los autores, individualizar células neoplásicas en una abundancia relativa de 1 por cada 10.000 células sanguíneas nucleadas. Es relevante destacar que las condiciones de señal versus ruido y límite de detección establecidos por los autores, les permitió reanalizar muestras históricas y comparar con hallazgos previamente reportados con un alto nivel de concordancia [43].

La versatilidad de los reporteros para IMC permitió que esta herramienta, haya sido utilizada para la detección de oligonucleótidos y ácidos nucleicos. Ya previamente se podían evaluar componentes nucleares a través de moléculas intercalantes del ADN o bien, la detección de alguna histona; sin embargo, los resultados de Schulz y colaboradores [45] plantean la posibilidad de estudiar de manera simultánea los niveles de ARN mensajeros (ARNm) y proteínas en muestras histológicas. La principal innovación de estos autores fue la modificación sobre el protocolo de RNAscope^®^ [46] para hibridación *in situ*, remplazando la sonda de detección por un oligonucleótido conjugado con un metal reportero para IMC. Terminada la hibridación, el tejido o muestra fue apta para procesamientos posteriores, tales como una inmunodetección múltiple de proteínas para IMC.

Para la validación de la nueva aplicación, se consideró concordancias entre hibidración *in situ* fluorescente (FISH) y la señal procedente del reportero metálico de la sonda, considerando la detección de los ARNm de genes de expresión constitutiva (POLR2A, PPIB y UBC) en células HeLa embebidas en parafina. La correlación entre métodos de detección estuvo entre 0,89 y 0,8 [45].

En términos muy generales, la verificación de poder realizar el análisis simultáneo de ácidos nucleicos y proteínas, se realizó en 70 casos de cáncer de mama considerando 16 blancos proteicos y 3 blancos de ARN. Para ello, se evaluó la correlación entre la abundancia de proteínas y ARN mensajeros para el receptor del factor de crecimiento epidérmico humano 2 (HER2) y citoqueratina 19 (CK19); sin embargo, los resultados no fueron concluyentes, pudiendo ser atribuido a algún mecanismo regulatorio no abordado en el diseño del experimento. De todos modos, la estrategia reportada permite la detección de ARNm y proteínas simultáneamente, permitiendo una caracterización detallada a nivel celular, fenotípico y funcional de células únicas en tejidos FFEP mediante IMC.

Finalmente, del punto de vista metodológico, varios autores han compartido generosamente sus experiencias y publicado sus resultados. Recientemente, un artículo resume las condiciones de tinción y manejo de anticuerpos para un panel de 40 biomarcadores, enfatizando que las características de la recuperación antigénica como elemento a considerar. Los autores comparan el pH del tampón de recuperación en condiciones estándar de temperatura y presión, indicando que para los anticuerpos seleccionados, el pH bajo (10 mM Citrato pH=6) tendría un efecto favorable respecto de utilizar un pH alto (10 mM Tris/1 mM EDTA pH=9), basado en resultados de IHQ individuales y detección con DAB. Después de evaluar 65 anticuerpos, el aporte de Ijsselsteijn y colaboradores [47] corresponde a una selección de 40 anticuerpos monoclonales, sus conjugados, sus condiciones de incubación y diluciones de trabajo. Desafortunadamente no se indican las concentraciones de cada uno de los stocks, no siendo posible homologar condiciones. No obstante, lo didáctico y explicativo del artículo, hace de éste, una pieza relevante al momento de diseñar un protocolo de IHQ para IMC.

## MIBI, el nuevo en el barrio

Dentro de las herramientas para obtención de imágenes mediante espectrometría de masa, la herramienta diseñada por Angelo y colaboradores [34] y mejorada posteriormente con el acoplamiento al análisis de tiempo de vuelo (TOF) [35], ha demostrado ser altamente reproducible y fiable para el análisis de muestras histológicas frescas y embebidas en parafina. Las primeras experiencias en imágenes por haz de iones multiplexados (MIBI, multiplexed ion beam imaging) fueron generadas a partir de células mononucleares de sangre periférica humana (CMSP), las cuales fueron marcadas en suspensión utilizando marcadores de superficie clásicos, tales como CD3, CD4, y CD8 para linfocitos T, CD14 principalmente para monocitos, CD19 para linfocitos B, y como marcadores genéricos de células inmunes CD45 y HLA-DR. Dos fracciones idénticas fueron generadas, una fracción celular fue embebida en silicona para su análisis por MIBI, y otra fue procesada para citometría de masas. El análisis mostró para ambas herramientas, una alta correlación en las intensidades de señal y recuento absoluto de elementos detectados para cada categoría, observando un rango dinámico de hasta 10.000 cuentas para el MIBI, y una varianza menor al 1% entre la citometría de masa y el análisis de imagen. Además, basado en la estrategia de tinción, MIBI permitiría el análisis de células aisladas y/o crecidas en suspensión sin necesidad de realizar un frotis o impronta, pudiendo ser estas células centrifugadas y embebidas en silicona u otro sustrato compatible. En ese contexto, tejidos FFEP han sido también analizados mediante MIBI, para ello, muestras de 5um de espesor han sido marcadas directamente con combinaciones de 10 anticuerpos [34] incialmente, y luego aumentado hasta 40 [35].

Cabe señalar que los resultados de análisis histológicos realizados sobre imágenes obtenidas mediante MIBI fueron congruentes con herramientas clásicas de IHQ. Angelo [34] probó que la modificación química del anticuerpo primario no altera su especificidad, generando señales en intensidad y ruido de fondo comparables a una tinción cromogénica. Por otro lado, el análisis automatizado y cuantitativo de imágenes mediante herramientas bioinformáticas resultó ser compatible, e incluso existiría una alta concordancias con herramientas actualmente disponibles y validadas para uso diagnóstico (por ejemplo QIA, q*uantitative image analysis FDA-approved*, en inglés), alcanzando una puntuación de H (H score) de 1,06.

De acuerdo a diferentes investigadores, las ventajas de MIBI son múltiples cuando se compara con una técnica de IHQ convencional. 1) más sensibilidad y mejor rango dinámico analítico, aumentando entre 100 y 1000 veces la razón señal versus ruido comparado contra una tinción de fluorescencia y una tinción cromogénica, respectivamente. 2) más especificidad, si bien depende del anticuerpo primario usado, gracias a la alta resolución analítica dada por el análisis de masa (fracción de Dalton) no se observa una superposición espectral entre reporteros adyacentes y la multiplicidad de dianas detectables. 3) un mejor aprovechamiento del material biológico escaso, los reporteros son altamente estables en el tiempo y las características de MIBI, las muestras pueden ser escaneadas en múltiples oportunidades con distintos niveles de resolución (entre 260 nm y 1um).

Más recientemente, MIBI ha demostrado ser útil en el estudio de cáncer de mama triple negativo con un extensivo panel de 36 blancos proteicos, permitiendo establecer que ciertas características fenotípicas de la célula están relacionados con la arquitectura del tejido, y su entorno adyacente [35]. Así los autores describen una heterogénea distribución de células PD-L1 positivas, tanto tumorales como no tumorales, la cual sería inter- e intra- pacientes, y además observaron una alta abundancia de células tumorales HLA-DR positivas en los márgenes del tumor y el estroma, lo cual estaría vinculada con mayor supervivencia [35].

## Aplicaciones innovadoras

### Modelos no-humanos

IMC parece ser más versátil de lo pensado, y ha sido empleada no sólo en tejido humano, sino también en tejido animal. Considerando todas las recomendaciones de validación de anticuerpos y condiciones de inmunodetección discutidas antes, Theil y colaboradores [48] establecieron un panel de 9 anticuerpos para la caracterización de los subgrupos linfocitarios en sangre y en tejido linfoide en monos Cynomolgus sometidos a regímenes de Ofatumumab a dosis equivalentes a las utilizadas en humanos [48]. Es meritorio reconocer la plasticidad de esta técnica y como lentamente se va insertando en estudios con mayor impacto en la salud humana. Ofatumumab es el primer anticuerpo monoclonal humano anti-CD20 en fase 3 de estudio clínicos para el tratamiento de la esclerosis múltiple.

### Fisiopatología renal

La necesidad de recrear la complejidad del tejido humano, normal y patológico, está aún vigente, y las iniciativas en esa dirección han ido aumentando significativamente. Singh y colaboradores [49] mediante un set de 23 anticuerpos y con una muy alta resolución, describió un atlas bidimensional del riñón humano, desde corteza a médula, y dada la sensibilidad, especificidad y multiplicidad de señales en el análisis, los autores fueron capaces de describir en detalle estructuras tubulares intermedias, no previamente estudiadas a ese detalle [49]. Adicionalmente, este atlas consideró el análisis de tejido patológico, y enfatizó en las poblaciones de células inmunes infiltrantes en condiciones de trasplante renal y nefritis. La validación de anticuerpos y protocolos fueron compartidos en la página web (Re)Building a Kidney (https://www.rebuildingakidney.org/) [50].

### HER2 y trastuzumab

Del punto de vista de la investigación del cáncer, IMC y otras tecnologías han aportado tremendamente al desarrollo de conocimiento, y quizás más al desarrollo de biomarcadores. Actualmente, trastuzumab es un anticuerpo terapéutico ampliamente utilizado en cáncer de mama, y cuyo punto de unión se ubica en el dominio extracelular de HER2 [51]. Una de las ventajas de IMC es que permite evaluar un mismo marcador desde dos perspectivas, de este modo [52], incluyó dos anticuerpos anti-HER2 en un mismo panel, cuyas especificidades están orientadas hacia un epitope en el dominio extracelular (ECD) y otro epitope hacia el dominio intracelular (ICD). Tras evaluar retrospectivamente biopsias de cáncer de mama, los casos que recurrieron después de adyuvancia con trastuzumab fueron aquellos con índices de positividad reducidos para HER2 ECD. Clínicamente, una razón ECD/ICD aumentada tendría una correlación con un tiempo libre de recurrencia mayor a 5 años, y una abundancia significativa de linfocitos citotóxicos CD8 positivos en la vecindad del tumor. Si bien los autores consideraron otros 16 anticuerpos en su estudio, ciertamente, la validación ortogonal entregada por Carvajal-Hausdorf y colaboradores, y la evidencia previamente reportada por el mismo grupo sobre el rol de los dominios de HER2 permiten sugerir que una inmunodetección convencional para uno o dos marcadores simultáneos gana valor, después de haber descartado aquellas otras variables con menor significancia.

### Estrategias de análisis

Respecto de las estrategias de análisis de imágenes, previamente la mayor parte de los autores han utilizado las herramientas de MCD™ viewer de Fluidigm [53], aplicación que permite identificar los niveles detectados por cada metal reportero además de su ubicación geográfica en el tejido; sin embargo, los principales análisis primarios y secundarios han sido desarrollados a través de las herramientas descritas por el grupo del Dr. Bernd Bodenmiller de la Universidad de Zurich en Suiza [54], [55], [56]. Estas aplicaciones están disponibles de manera abierta, y permiten además de cuantificar la señal, segmentarla en áreas de tejido, conferir atributos y definir el fenotipo celular para un análisis de multiparamétrico adecuado.

Recientemente, el grupo de David Rimm de la Universidad de Yale en EE.UU., reportó el uso de AQUA™ (Navigate BioPharma Inc) para el análisis primario de imágenes colectados mediante IMC [57]. Este software ha sido ampliamente utilizado por este grupo para el análisis cuantitativo de fluorescencia en contexto de máscaras de segmentación asociado a pixeles [58, 59]. En el trabajo de Martínez-Morilla y colaboradores se desarrolló en lesiones de melanoma maligno, donde el software AQUA toma los pixeles de las áreas reactivas para el Intercalador de ADN (Ir191/193) y para HMB45+S100 para la construcción de las máscaras para tejido y tumor, respectivamente. De manera similar, el perfilamiento digital del tejido y otras células ocurre basado en la densidad de pixeles con cuentas para cada uno de los reporteros. Esta simplificación en el análisis permitió a los autores establecer a partir de un panel con 26 anticuerpos, un número de 12 marcadores con significativa asociación con sobrevida libre progresión, y de 7 anticuerpos relacionados con sobrevida global. Luego de una validación mediante análisis de RNA mensajeros y detección fluorescente simple, beta-2-microglobulina resultó un prometedor biomarcador relacionado con sobrevida en casos de melanoma metastásico e inmunoterapia [57].

En la actualidad, otros actores privados han ido contribuyendo en el desarrollo de herramientas informáticas más amigables con el usuario, tales como Indica labs (HALO^®^) y Visiopharm^®^.

### Validación de múltiples componentes

Recientemente, se evaluó la capacidad de MIBI para analizar múltiples tipos de tumores sólidos, en su mayoría no relacionados del punto de vista del órgano o el tejido lesionado. El estudio consideró 15 neoplasias, incluyendo adenocarcinomas, carcinomas escamosos y neoplasias hematológicas [60]. De la misma manera que sus precedentes, los autores evaluaron la especificidad y sensibilidad de sus anticuerpos mediante IHQ, considerando controles de positividad y negatividad endógenos dentro de la misma sección de tejido analizado. Un panel con 15 anticuerpos fue utilizado para inmunodetectar secciones de 1 mm de tejido, y la captura de imágenes se realizó en campos ópticos de 0.25 mm^2^ (0.5 µm por pixel). Un mérito de este trabajo, fue incluir una herramienta estadística simple de tipo “dejar uno fuera” (leave-one-out, LOO) para la verificación de las señales obtenidas y la no contaminación entre reporteros cercanos. Los autores realizaron múltiples tinciones excluyendo uno a uno cada anticuerpo del panel principal, de ese modo compararon los resultados obtenidos entre el panel completo (15 anticuerpos) y sucesivos paneles dejando uno de los anticuerpos fuera (14 anticuerpos). Tras 8 paneles de comparación, se realizaron análisis de regresión lineal con una correlación R2=0.99–1.00, indicando que no existía interferencia entre fuentes de señal. Bajo esa premisa, los autores realizaron análisis de segmentación tisular y fenotipo celular en cada uno de las neoplasias seleccionadas, confirmando la alta heterogeneidad del componente inmune infiltrante, tanto en abundancia y distribución que se observa entre tipos tumorales y entre individuos con un mismo tipo de lesión [60].

## Conclusiones

Considerando las similitudes entre la tecnología de IMC (incluyendo MIBI) y la inmunohistoquímica clásica, así también, los fundamentos de las detecciones multiplexadas, es necesario reconocer las ventajas y desventajas genéricas que éstos pueden presentar, y que serán determinantes al momento de su implementación y uso (Tabla 3).

No obstante, tanto IMC como MIBI, son herramientas que permiten la colección de imágenes complejas con resolución espacial y la cuantificación de múltiples componentes de manera simultánea. Destacable es que ambas estrategias son comparables con técnicas histológicas clásicas en términos de especificidad y sensibilidad. Del punto de vista de cuantificación, precisión y reproducibilidad, tanto IMC y MIBI han probado ser comparables y robustos en distintas matrices y distintos blancos de análisis, no siendo una dificultad la complejidad o simpleza del panel de anticuerpos, salvo por respectar la individualidad de cada reportero.

## Perspectivas

La compresión de los procesos fisiológicos en condiciones normales y patológicas requiere disponer de una visión general en la abundancia de biomarcadores, así como su distribución en el espacio. En condiciones patológicas complejas como las neoplasias, la correlación entre genotipo y fenotipo de los distintos componentes celulares y su entorno son vitales para la asignación de terapias, medición de beneficio, e incluso mitigar fracasos terapéuticos. La captura de imágenes de componentes múltiples a través de IMC, MIBI u otra herramienta ha sido ampliamente validada y verificada por diferentes investigadores, resaltando siempre la necesidad de recurrir a una técnica de referencia para anticipar desviaciones. Esta revisión pretende entregar al lector una visión panorámica de las alternativas que puede implementar en su investigación, disponiendo de literatura seleccionada para reducir la curva de aprendizaje y obtención de resultados.

**Tabla 3:** Ventajas y Desventajas entre herramientas para análisis de biomarcadores en tejidos fijados y embebidos en parafina.

Herramienta	Usode tejido		Blancos posibles		Reporteros	
	Ventaja	Desventaja	Ventaja	Desventaja	Ventaja	Desventaja
Inmunohistoquímica clásica	Preserva la lámina para multiples lecturas	Múltiples láminas en estudios complejos	1 a 2 blancos (más contraste nuclear). El marcador puede ser analizado por múltiples observadores	Restringido número de combinaaones, tanto por método de detectión como por localizatión de lamarca	Mayormente depósitos cromogénicos insolubles y estables en el tiempo	Umitado número de opciones (enzimas, conjugados y cromógenos)
Immunodetectión fluorescente multiplexada	Menor número de láminas en estudios complejos	Láminas pueden ser preservada para segundas lecturas	Hasta 9 blancos según plataforma. Permite múltiples señales en localizaciones similares	Requiere validación exhautiva ycostosa	Múltiples combinaciones según proveedor. Permite uso de amplificadores de señal	Riesgo de fotoblanqueo y superpositión espectral de señales fluorescentes cercanas
Immunodetectión multiplexada tipo IMC	Menor número de láminas en estudios complejos	Destructión del tejido. Mayormente no permite segundas lecturas	Hasta 60 blancos según plataforma. Permite múltiples señales en localizaciones similares	Requiere validación exhautiva ycostosa	Múltiples combinaciones según proveedor. Limitado riesgo de superposiciónde reporteros	Accesible comerdalmente o por preparación propia. Reactivo muy costoso. Limitado uso de amplificadores de señal
